# Lessons learned from COVID-19 for the post-antibiotic future

**DOI:** 10.1186/s12992-020-00623-x

**Published:** 2020-10-08

**Authors:** Lindsay A. Wilson, Susan Rogers Van Katwyk, Patrick Fafard, A. M. Viens, Steven J. Hoffman

**Affiliations:** 1 Global Strategy Lab, York University/University of Ottawa, 4700 Keele Street, 2120 Dahdaleh Building, Toronto, ON M3J 1P3 Canada; 2 Graduate School of Public & International Affairs, Faculty of Social Science, University of Ottawa, Ottawa, ON Canada; 3 School of Global Health, York University, Toronto, ON Canada; 4 Dahdaleh Institute for Global Health Research, Faculty of Health and Osgoode Hall Law School, York University, Toronto, ON Canada; 5 Department of Global Health and Population, Harvard T H Chan School of Public Health, Harvard University, Boston, MA USA; 6 Department of Health Research Methods, Evidence, and Impact and McMaster Health Forum, McMaster University, Hamilton, ON Canada

**Keywords:** COVID-19, Antimicrobial resistance, Emergency preparedness, International cooperation

## Abstract

**Introduction:**

COVID-19 has rapidly and radically changed the face of human health and social interaction. As was the case with COVID-19, the world is similarly unprepared to respond to antimicrobial resistance (AMR) and the challenges it will produce. COVID-19 presents an opportunity to examine how the international community might better respond to the growing AMR threat.

**Main body:**

The impacts of COVID-19 have manifested in health system, economic, social, and global political implications. Increasing AMR will also present challenges in these domains. As seen with COVID-19, increasing healthcare usage and resource scarcity may lead to ethical dilemmas about prioritization of care; unemployment and economic downturn may disproportionately impact people in industries reliant on human interaction (especially women); and international cooperation may be compromised as nations strive to minimize outbreaks within their own borders.

**Conclusion:**

AMR represents a slow-moving disaster that offers a unique opportunity to proactively develop interventions to mitigate its impact. The world’s attention is currently rightfully focused on responding to COVID-19, but there is a moral imperative to take stock of lessons learned and opportunities to prepare for the next global health emergency.

## Introduction

COVID-19 has radically changed the face of human health and social interaction in the span of a few months. This should not come as a surprise: health researchers have long warned that the world’s lack of pandemic preparedness would have catastrophic implications. We are similarly unprepared to deal with the growing threat of antimicrobial resistance (AMR) and the challenges it will produce [1, 2]. The impact of AMR can already be felt through rising morbidity, mortality, and healthcare costs associated with infections that were previously considered treatable [2]. As COVID-19 reveals the cracks in emergency responses around the world, it also offers important opportunities to examine how our health systems, social and economic policies, and global institutions might better respond to the growing AMR threat.

## Health system implications

COVID-19 has had enormous consequences for health systems around the world, overwhelming even some systems considered well-prepared for pandemic emergencies. This pressure has forced governments and healthcare providers to make difficult decisions about prioritizing medical care and cancelling all but the most urgent procedures [3]. Similar quandaries about shifting priorities and resource scarcity will arise as the pressure that AMR places on healthcare systems increases, including longer hospitalizations, longer treatment times, and increased risk associated with common surgical procedures [4]. Increasing drug resistance will push these boundaries further as hospital outbreaks and limited bed space necessitate a proactive response to deep ethical questions about prioritization of care, both in terms of what treatments and procedures are offered and who will receive care.

These ethical dilemmas are often more acute in low- and middle-income countries (LMICs), where scarce resources limit capacity to address additional demands on the health system. In LMICs, access to effective antimicrobials is already inadequate [5], yet rates of AMR are anticipated to increase 4–7 times more quickly [2]. Furthermore, COVID-19 has likely accelerated the pace at which AMR-related threats are growing, due to high rates of (often inappropriate) antibiotic prescribing for patients with COVID-19, interruptions in treatment for patients with chronic conditions, and widespread use of antimicrobial disinfecting agents by individuals and institutions [6]. All of these factors increase the risk of AMR and greatly impact AMR stewardship. Learning from the health system demands caused by COVID-19, health system actors should take measures to prepare for the rising health system burden caused by AMR and develop contingency plans to minimize harm. This includes: proactively strengthening health systems through investments in expanded hospital capacity for both patients and laboratory testing for drug resistance, ensuring adequate antimicrobial stewardship training for personnel, and securing adequate supplies of antimicrobials and PPE to treat patients. The prevention of resistant infections will also be critical, and should include proactive investment in health promotion and water, sanitation, and hygiene (WASH) that will prevent the development of resistant infections both at home and in-hospital [2].

## Economic and social implications

COVID-19 has also had an unprecedented impact on national economies, resulting in widespread unemployment, depletion of personal savings, and economic downturns. Massive AMR-related losses are predicted due to lost productivity as sick leaves and long recovery times prevent people from working [2]. The labour market impact of AMR is expected to have large cumulative effects on the global economy, especially in service industries that rely on human interaction [2, 4]. Early action to strengthen social safety nets and prepare for potential impacts on pensions, investments, and small businesses will have sizeable benefits in the future [2].

The current pandemic also shows that the socioeconomic impacts of health emergencies are not distributed equitably. School and childcare closures in response to COVID-19 have forced millions of people to balance working from home with caring for family members. As women continue to provide the majority of informal care within families, this has substantial impacts on their careers and work opportunities, often forcing them to drop out of the workforce [7]. Men and women are often differentially affected by disease outbreaks, and women’s needs are frequently neglected in the discourse surrounding outbreaks. Historically, resources have been diverted from sexual, reproductive, and maternal health into emergency response procedures [7]. A comprehensive AMR approach that appropriately addresses the differential biological and social impacts of AMR should include equitable and gender-appropriate access to healthcare, strengthened worker protections, and alternative arrangements for child and senior care in cases where regular provision may be disrupted. This may involve greater support from employers for flexible working arrangements and increased social safety nets to provide support for medical and caregiver leaves of absence. Furthermore, women’s voices should be meaningfully included in analyses of the gendered impacts of AMR.

## Global political implications

The breakdown of global solidarity during COVID-19 has underlined the fragility of international rules-based cooperation. During the pandemic, many countries ignored the World Health Organization’s guidance and violated the legally-binding *International Health Regulations* (2005) by barring exports of medical supplies, imposing harsh travel restrictions, and infringing human rights obligations [8] in order to prevent the virus from spreading within their own borders.

AMR’s global impacts will require a cohesive and enforceable global response. COVID-19 has demonstrated that current approaches to international cooperation are not fit-for-purpose when it comes to a global pandemic, and the consequences are being felt worldwide. The international community should re-examine the feasibility of a coordinated global response to AMR under existing institutions and implement any necessary changes to promote solidarity and prevent governments from introducing harmful restrictions in the name of preventing the spread of resistant pathogens.

One approach that may facilitate effective collective action is the adoption of a unifying global target against AMR that rallies support from individuals, businesses, civil society actors, and governments. The COVID-19 call to “flatten the curve” has acted as a memorable, mobilizing target, encouraging people to slow the rate of new infections and avoid overwhelming the healthcare system. Drawing from this experience, the international community should proactively find a similarly compelling rallying-cry in order to offer the best chance of an effective coordinated response to AMR across sectors [9]. While no equivalent rallying cry currently exists in the context of AMR, initiating this global discussion will help the global community mobilize support and resources to promote further buy-in for other interventions, including those that are specific to AMR (e.g., greater, coordinated investment in novel treatments for resistant infections; clear and feasible prescribing practices) and those that address AMR indirectly while working toward other targets, such as the Sustainable Development Goals (e.g., improved access to clean water and sanitation). Discussions of global targets or agreements for AMR must take local context and circumstances into account when determining national commitments, and any commitments should be reviewed frequently to ensure they are feasible and effective [10].

## Conclusion

COVID-19 has illustrated the ease with which infectious diseases transcend borders and spread in our interconnected world. Even the highest performing healthcare systems are only as prepared as their most vulnerable counterparts in our global community [11]. The world’s attention is currently rightfully focused on responding to COVID-19, but there is a moral imperative to take stock of lessons learned and opportunities to prepare for the next global health emergency. The insights from COVID-19 should fuel a research agenda focused on avoiding a future AMR pandemic—often characterized as a “slowly-emerging disaster” [12]—that could prove even more costly than COVID-19. By heeding the lessons of COVID-19, the worst-case scenario for AMR may be avoided.

## Data Availability

Not applicable.
